# The Week's Work

**Published:** 1911-06-10

**Authors:** 


### THE WEEK'S WORK.

### THE HOSPITAL SUNDAY NUMBER.

This year we publish as usual a helpful and instructive Hospital Sunday Special Number, which contains several important articles to which we would draw our readers' attention. In "The Needs of To-day" the general plea of the hospitals is set forth; the detailed figures given in the article dealing with "The Income of our Hospitals and Dispensaries" show clearly the proportionate income derived from various sources, and the shares which the dead and the living hands have in keeping up the voluntary institutions in this country. A correspondent from Hong Kong contributes an instructive article showing how hospitals help the Englishman abroad. A fourth paper gives cogent answers to the question, "Why should I help the Hospitals?" a fifth deals with the reasons for, and causes of, increased expenditure in hospitals; and the sixth and concluding article explains the usefulness of convalescent homes as adjuncts to hospitals. Details of the special needs of London institutions are given, together with statistics. The number is one that should be distributed freely, for it contains much that is of practical value and interest to the public on Hospital Sunday.

### THE UNITED HOSPITAL RALLY.

The magnificent rally of the hospitals to the call of the British Hospitals' Association last week, and the great distances from which many representatives came at the shortest notice, show the need which the Association fills, especially at moments of crisis like the present. Only a little more effort is needed to make the Association all-embracing, and we welcome the letter of Mr. Thies, its honorary treasurer, calling upon any hospitals who are not yet represented upon it, to become so without delay. The opportunity of impressing a united hospital opinion upon the Chancellor must not be thrown away, and it must be remembered that the British Hospitals' Association so far has been the only representative body which has fought for the voluntary hospitals at all, and has supplied the only collective voice which they have been able to raise on the provisions of the Insurance Bill which at present threaten the voluntary system so deeply.

### A FALSE RUMOUR AT CHICHESTER.

The rumour afloat that the Chichester General Infirmary was going to be closed during July, when its reconstruction will be taken in hand, has been officially contradicted. We learn, in the words of Major Chauncy, the Chairman of the Board of Management, that probably the whole eastern half of the building will be closed. This means, generally speaking, that the staff will be left unhindered, while the principal portions of the reconstruction scheme, namely, the new theatre and nurses' home, are being built. When this part is finished an attempt will be made to carry on the work from it, but as the boiler-house and kitchen will then have to be placed in the workmen's hands, temporary quarters will probably have to be found for casualties in the city. It will not have been forgotten that The Hospital of July 10, 1910 (page 418), reported the Chichester Infirmary old buildings to be "possibly the best specimen extant of the old H-plan of construction." We deprecated most strongly the appeal for £20,000, which the Bishop of Chichester was induced to issue last autumn, as extravagant in the extreme. It is necessary only to add that it was the sum of money then asked for and not the new theatre and sanitary block that called forth this criticism.

### THE NEW CASUALTY WING AT DEWSBURY.

We are glad to note the opening last week of the last of the series of improvements that have been undertaken recently at the Dewsbury General Infirmary. It will be remembered that the bequest of over £7,000 from the late Mrs. Cardwell enabled certain extensions to be undertaken and the endowment fund to be increased. Of the former the latest addition is the casualty wing, which Major Walker, the Mayor, has just declared open, and which has cost £1,200. It consists of a large casualty room, a room for the holding of inquests and committee meetings, and two consulting rooms. Its flat roof is to be used for convalescent cases, being in easy reach of the men's ward. Mr. Edward Hemingway, the secretary, is to be congratulated on the successful way in which his hospital is growing.

### MEETING OF THE LONDON PROVIDENT DISPENSARIES COUNCIL.

The Duke of Devonshire has been elected Chairman of the London Provident Dispensaries Council which met last week to discuss the probable effect of the medical clauses of the State Insurance Bill on the future of provident dispensaries. It is worth emphasising, perhaps, that the dispensaries provide medical treatment and medicine on an insurance basis for over 110,000 persons of the industrial classes and possess a medical staff of more than 320 general practitioners. The position of the Provident Dispensaries under the Bill is one that has been pondered most anxiously by all those interested in the continuation of the voluntary system.

### WHAT FLAG SHOULD A HOSPITAL FLY?

Some months ago a correspondent wrote to inquire whether a hospital which had received a gift of a flag-pole and an assortment of flags was entitled to fly the Union Jack or not. The question raised an interesting point, and we have accordingly been at some pains to investigate the matter. While abroad the rights of hospitals to bear arms, to fly flags, and to certain other privileges are very exactly defined, in this country they appear to be left entirely to such definition, or want of definition, as custom may give. In Russia, for example, hospitals controlled by the State may fly the State flag; on certain days of the year the Imperial standard may be flown, as the Czar is the patron of every

State hospital. Private clinics—which in Russia correspond to our voluntary hospitals—are not allowed to fly any flag which has a national significance; hospitals belonging to specified " colonies " (English, French, or German) may fly their respective flags by courtesy. In Germany the regulations are similar, with the exception that the Imperial standard may not be used, but only the national flag. In Italy and France no special exceptions obtain: any hospital may, if it wishes, fly the national flags. In the United States, specific resolutions of Congress allow every public institution to fly the Commonwealth flag: private hospitals may fly the flag by courtesy or by the right sanctioned by custom. We shall add further points next week.

### THE MANSFIELD HOSPITAL EXTENSION SCHEME.

It is satisfactory to note that the recent recommendation for extension made by the Management Committee of Mansfield Hospital, Nottingham, has been adopted. Immense impetus to it has been given by the announcement of the gift of £1,000 from the Duke of Portland, who is the hospital's President this year. On Thursday last week Mr. H. E. Hollins, Chairman of the hospital, explained the position at a meeting which Alderman Taylor, the Mayor of Mansfield, had convened. Mr. Hollins said that they had at present an accident hospital in Mansfield with forty beds, and a medical hospital with sixteen beds at Mansfield Woodhouse. The latter branch the Board has been empowered to sell, as The Hospital indeed recorded on August 27, 1910 (page 657), and, with the encouragement that the Duke of Portland's support has given, it is hoped there will be little difficulty in raising the £10,000 which is the estimated cost of the new enlargement scheme. This scheme will add two isolation wards and give better accommodation to the domestic staff, a section the interests of which have been looked after adequately only of recent years. The industrial development of Mansfield has increased inevitably the demand on the hospital's beds, a demand, we regret to add, which does not seem to have been recognised by the colliery owners of the neighbourhood. Mr. Hollins, however, is going the right way to work, and we shall hope to be able to report the success of his efforts.

### THIS WEEK'S DRUG MARKET.

Business in the drug market has been quiet, as is usually the case at holiday times. The quinine market, which has been so quiet for a long period, shows signs of activity, and a fair business has been done at slightly higher prices. The tendency to higher prices is due to the fact that the quantity of cinchona bark offered at this month's public auctions in Amsterdam is smaller than usual. The price of eserine has been advanced considerably. Carbolic acid tends dearer. Oil of male fern is lower in price. Cod-liver oil still tends lower in price; in the Finmarken district there has been a heavy yield of oil which to a very considerable extent makes up for the small yield in the Lofoten district. Tartaric and citric acids are in good demand, owing to the hot weather, and prices tend to advance. An advance in the price of cascara sagrada is considered probable. The price of olive oil is not maintained quite so firmly, and a small decline in value is not improbable. Turpentine is declining in value. Opium and its alkaloids are unchanged.

